# COVID-19 prevalence and predictors in United States adults during peak stay-at-home orders

**DOI:** 10.1371/journal.pone.0245586

**Published:** 2021-01-22

**Authors:** Robert Morlock, Amy Morlock, Martha Downen, Sonali N. Shah

**Affiliations:** 1 YourCareChoice, Ann Arbor, Michigan, United States of America; 2 Acumen Health Research Institute, Ann Arbor, Michigan, United States of America; 3 Downen Consulting Group, LLC, Rye, New York, United States of America; 4 Independent Scholar, Freehold, New Jersey, United States of America; Dasman Diabetes Institute, KUWAIT

## Abstract

**Background:**

Early recognition of COVID-19 cases is essential for effective public health measures aimed at isolation of individuals infected with severe acute respiratory syndrome coronavirus 2 (SARS–COV-2). The objective of this study was to describe characteristics, self-reported symptoms, and predictors of testing positive for SARS-CoV-2 infection in a community-based sample.

**Methods and findings:**

This was a cross-sectional nationwide survey of adults in the US conducted between April 24 through May 13, 2020. The survey targeted a representative sample of approximately 5,000 respondents. The rate of COVID-19 cases and testing, most frequently reported symptoms, symptom severity, treatment received, impact of COVID-19 on mental and physical health, and factors predictive of testing positive were assessed. Most of the 5,203 participants (85.6%) reported no COVID-19-like symptoms. Of the 747 (14.5%) participants reporting COVID-19-like symptoms, 367 (49.1%) obtained a diagnostic test. Eighty-nine participants (24.3%) reported a positive COVID-19 test result, representing 1.7% of the total sample. For those testing positive, the most common symptoms were dry cough, fever, and shortness of breath/difficulty breathing. Those who tested positive were more likely to report greater symptom severity versus those who tested negative. Severe dry cough, new loss of taste or smell, trouble waking up, living with someone experiencing symptoms, recent international travel, respiratory issues, and reporting ethnicity of Black or African American were predictive of testing positive.

**Conclusions:**

This study assessed the impact of COVID-19 using community-level self-reported data across the US during the peak of most stay at home’ orders. Self-reported symptoms and risk factors identified in this study are consistent with the clinical profile emerging for COVID-19. In the absence of widespread testing, this study demonstrates the utility of a representative US community-based sample to provide direct-reported symptoms and outcomes to quickly identify high-risk individuals who are likely to test positive and should consider taking greater precautions.

## Introduction

The first case of the COVID-19 in the US was confirmed by the Centers for Disease Control and Prevention (CDC) on January 22, 2020 [1]. The World Health Organization (WHO) subsequently declared COVID-19 a global pandemic on March 11, 2020 [2]. By mid-March, the US announced coronavirus guidelines and many states issued stay-at-home orders early in the pandemic [3].

Access to testing has been a barrier to understanding the true prevalence of COVID-19 cases in the community. Early in the pandemic, access to COVID-19 tests was limited to individuals who met specific criteria, including healthcare professionals, international travel to select high-risk countries, hospitalization with COVID-19-like symptoms, or individuals who were in close proximity to someone diagnosed with COVID-19 [4]. During this phase, total cases and deaths reported per day were primarily based on individuals who were hospitalized and were aggregated by states. Individuals who did not seek hospital care and remained in the community would have been underrepresented in these counts.

The incidence and mortality rates associated with SARS-CoV-2 infection have been difficult to calculate given the true numerator of the total number of infections is unknown. Studies suggest the number of confirmed cases may have underestimated the number of COVID-19 cases by as much as 10-fold [5]. The incidence may have been even greater earlier in the pandemic given limited access and prioritization of testing for select groups. Therefore, the full burden of COVID-19 has likely been underestimated. While previous studies have largely focused on characterizing patients who are hospitalized with COVID-19, the symptom profile for individuals who test positive for SARS-CoV-2 in the community is less well known.

The aim of this study was to better understand the prevalence of COVID-19 and testing rates in the community by conducting an online survey of a representative community-based sample of adults across the United States. Further data was collected on those participants who reported receiving a COVID-19 test to compare the most frequently reported symptoms, severity of symptoms, and medication management by positive and negative test results. Lastly, multivariate logistic regression analysis was conducted to identify key factors that may be predictive of a positive COVID-19 test outcome.

## Methods

### Data collection

Data was collected through an on-line cross-sectional survey of adults (18 years or older) in the US. The survey was conducted in accordance with Acumen Health Research Institute's (AHRI) established survey procedure. A random stratified sampling framework ensured a community-based sample with a demographic composition representative of the US adult population by region, gender, age, and race, according to the US Census (US Census American Community Survey 5-year estimate, 2011–2015). To participate in the study, respondents were required to be 18 years old or older, reside in the United States, and confirm their voluntarily agreement to participate (participants were informed they could leave the survey at any time). The survey was open to the general population and not restricted to patients hospitalized with COVID-19. Participants were recruited through AHRI’s online research panels.

Survey fielding from April 24 through May 13, 2020 targeted approximately 5,000 respondents. Multiple quality control processes integrated throughout data collection, including digital fingerprinting technologies that validate unique respondents, ensured study data was comprised of non-fraudulent respondents. This survey was deemed Institutional Review Board-exempt as all responses were anonymized, aggregated, and could not be related back to the participants.

### Patient cohorts

Participants were stratified into four main cohorts based on response to the question: Which statement about COVID-19 best describes you?: I tested positive for COVID-19 and have recovered or I tested positive for COVID-19 and am recovering; I thought I had COVID-19 but the test was negative; I thought I had COVID-19 but was not tested or I think I have COVID-19 but have not been tested; I do not think I have or have had COVID-19. Cohorts were adults who reported they:

- Never had COVID-19 (assumes no test) (COVID Not-suspected Cohort)
- Have or had COVID-19 and received a diagnostic test (COVID Suspected Cohort)
  ○ Have or had COVID-19 and tested positive (Positive Cohort)
  ○ Have or had COVID-19 and tested negative (Negative Cohort)
- Have or had COVID-19 without test confirmation (Untested Cohort)

All participants completed survey questions on demographics, comorbid conditions (used to calculate the Charlson Comorbidity Index [CCI]), health-related quality of life (HRQoL), economic/financial, mental, and physical impacts of the COVID-19 pandemic, and concerns related to the COVID-19 pandemic. HRQoL was measured using the Patient-Reported Outcomes Measurement Information System (PROMIS^©^) global mental health (GMH), and global physical health (GPH) scales. PROMIS^©^ GMH and GPH results were reported as standardized T-scores with general population norm scores of 50 with a standard deviation (SD) of 10, of which a higher score indicated better health [6,7].

Participants who reported they have or had COVID-19 (Positive Cohort, Negative Cohort, or Untested Cohort) were asked additional questions about COVID-19 symptoms, healthcare-seeking behaviors, factors associated with increased risk of contracting COVID-19, and absenteeism and presenteeism due to their COVID-19 illness. Symptom severity data was collected for the symptoms of shortness of breath/difficulty breathing, dry cough, and fatigue/tiredness. Treatment management data was collected for those who reported healthcare-seeking behavior (ie, seeing or calling a physician, going to the emergency room or urgent care, or going to the hospital and/or spending one or more nights in the hospital).

Participants completed a comorbidity checklist that was used to calculate the Charlson Comorbidity Index (CCI) score. A higher total index score (score range 0–27) indicates greater comorbid burden [8].

### Statistical analysis

An inferential test of proportions was carried out to describe differences in demographic, clinical, and symptom characteristics for those with positive vs negative COVID-19 tests. Chi-square tests were conducted to compare the distribution of categorical variables, while the Wilcoxon rank-sum test was used for continuous variables. To determine the predictors of testing positive for COVID-19, a backward stepping logistic model was constructed. The dependent variable (positive COVID-19 test) was modeled as a function of age, sex, race, weight/height, smoking status, international travel indicator, domestic travel indicator, living with someone that may have/had COVID-19 indicator, COVID-19 symptoms, and COVID-19 symptom severity and comorbidities. Regression coefficients (or their transformation, e.g., odds ratios [ORs]) with 95% confidence intervals and associated *p*-values are reported for predictors that are significant. A *P*-value <0.05 was considered statistically significant.

## Results

### Sample population and demographics

The survey was fielded to a general US adult population. Of the 6,161 potential participants, 5,203 (84.4%) completed the survey and were retained for analysis. The demographic composition of the sample was representative of the US adult population by region, gender, age and race, according to the US Census (Table 1). The survey was not restricted to treatment setting and only 75 respondents indicated that they had been hospitalized for their symptoms.

**Table 1:** Self-reported characteristics of study participants by cohort.

	Total Sample Population (N = 5,203) No. (%)	Positive Cohort (n = 89) No. (%)	Negative Cohort (n = 278) No. (%)	Untested Cohort (n = 380) No. (%)	COVID Not-Suspected Cohort (n = 4,456) No. (%)	P-value
Male	2,592 (49.82)	60 (67.42)	204 (73.38)	172 (45.26)	2,156 (48.38)	<0.001
Age, mean (SD)	47.48 (16.02)	37.37 (10.30)	38.86 (9.90)	43.17 (14.67)	48.58 (16.24)	<0.001
Age						
18 to 24 years	409 (7.86)	9 (10.11)	17 (6.12)	38 (10.00)	345 (7.74)	<0.001
25 to 34 years	864 (16.61)	30 (33.71)	64 (23.02)	82 (21.58)	688 (15.44)	
35 to 44 years	1,153 (22.16)	33 (37.08)	131 (47.12)	106 (27.89)	883 (19.82)	
45 to 54 years	884 (16.99)	9 (10.11)	45 (16.19)	58 (15.26)	772 (17.32)	
55 to 64 years	895 (17.20)	7 (7.87)	16 (5.76)	60 (15.79)	812 (18.22)	
65 to 74 years	844 (16.22)	1 (1.12)	5 (1.80)	31 (8.16)	807 (18.11)	
75 years or more	154 (2.96)	(0.00)	(0.00)	5 (1.32)	149 (3.34)	
Ethnicity						
Asian Only	348 (6.69)	4 (4.49)	9 (3.24)	25 (6.58)	310 (6.96)	0.001
Black or African American Only	523 (10.05)	18 (20.22)	23 (8.27)	23 (6.05)	459 (10.30)	
White Only	4,000 (76.88)	61 (68.54)	235 (84.53)	296 (77.89)	3,408 (76.48)	
Other or Mixed	282 (5.42)	5 (5.62)	9 (3.24)	30 (7.89)	238 (5.34)	
Prefer not to say	50 (0.96)	1 (1.12)	2 (0.72)	6 (1.58)	41 (0.92)	
Hispanic Origin	364 (7.00)	16 (17.98)	29 (10.43)	45 (11.84)	274 (6.15)	<0.001
Region						
Northeast	932 (17.91)	23 (25.84)	82 (29.50)	85 (22.37)	742 (16.65)	<0.001
Midwest	1,194 (22.95)	16 (17.98)	50 (17.99)	95 (25.00)	1,033 (23.18)	
South	1,892 (36.36)	32 (35.96)	79 (28.42)	109 (28.68)	1,672 (37.52)	
West	1,185 (22.78)	18 (20.22)	67 (24.10)	91 (23.95)	1,009 (22.64)	
Weight						
Under weight	263 (5.05)	14 (15.73)	16 (5.76)	28 (7.37)	205 (4.60)	<0.001
Normal weight	2,158 (41.48)	37 (41.57)	148 (53.24)	143 (37.63)	1,830 (41.07)	
Slightly overweight	1,607 (30.89)	22 (24.72)	77 (27.70)	124 (32.63)	1,384 (31.06)	
Moderately overweight	798 (15.34)	13 (14.61)	24 (8.63)	56 (14.74)	705 (15.82)	
Significantly overweight	377 (7.25)	3 (3.37)	13 (4.68)	29 (7.63)	332 (7.45)	
Charlson Comorbidity Index, mean (SD)	0.40 (0.96)	1.02 (1.78)	0.53 (1.18)	0.36 (0.86)	0.38 (0.92)	<0.001
Hypertension	1,231 (23.66)	23 (25.84)	46 (16.55)	77 (20.26)	1,085 (24.35)	0.009
Diabetes (Type I or Type II) without complications	624 (11.99)	21 (23.60)	49 (17.63)	33 (8.68)	521 (11.69)	<0.001
Chronic lung disease, chronic bronchitis or emphysema	246 (4.73)	6 (6.74)	5 (1.80)	19 (5.00)	216 (4.85)	0.098
Heart attack	121 (2.33)	5 (5.62)	7 (2.52)	11 (2.89)	98 (2.20)	0.161
Stroke or transient ischemic attack	95 (1.83)	3 (3.37)	6 (2.16)	5 (1.32)	81 (1.82)	0.591
Congestive heart failure	94 (1.81)	5 (5.62)	9 (3.24)	6 (1.58)	74 (1.66)	0.011
Mild liver disease, hepatitis, cirrhosis	74 (1.42)	5 (5.62)	4 (1.44)	13 (3.42)	52 (1.17)	<0.001
Diabetes with chronic complications	68 (1.31)	4 (4.49)	7 (2.52)	1 (0.26)	56 (1.26)	0.004
Peripheral vascular disease	49 (0.94)	4 (4.49)	3 (1.08)	7 (1.84)	35 (0.79)	0.001
Moderate or severe liver disease, hepatitis, cirrhosis	47 (0.90)	5 (5.62)	7 (2.52)	5 (1.32)	30 (0.67)	<0.001
Resource Utilization						
Physician visit or telemedicine	359 (48.06)	55 (61.80)	187 (67.27)	117 (30.79)	NA (NA)	<0.001
Emergency room or urgent care	179 (23.96)	41 (46.07)	101 (36.33)	37 (9.74)	NA (NA)	<0.001
Hospitalization	75 (10.04)	24 (26.97)	47 (16.91)	4 (1.05)	NA (NA)	<0.001
Antibody Testing	N = 407 (7.82)	n = 56 (62.92)	n = 141 (50.72)	n = 21 (5.53)	n = 189 (4.24)	
Tested positive	88 (21.62)	36 (64.29)	22 (15.60)	10 (47.62)	20 (10.58)	<0.001
Tested negative	298 (73.22)	15 (26.79)	116 (82.27)	7 (33.33)	160 (84.66)	
Not sure	21 (5.16)	5 (8.93)	3 (2.13)	4 (19.05)	9 (4.76)	

SD: Standard deviation.

**Fig 1:**
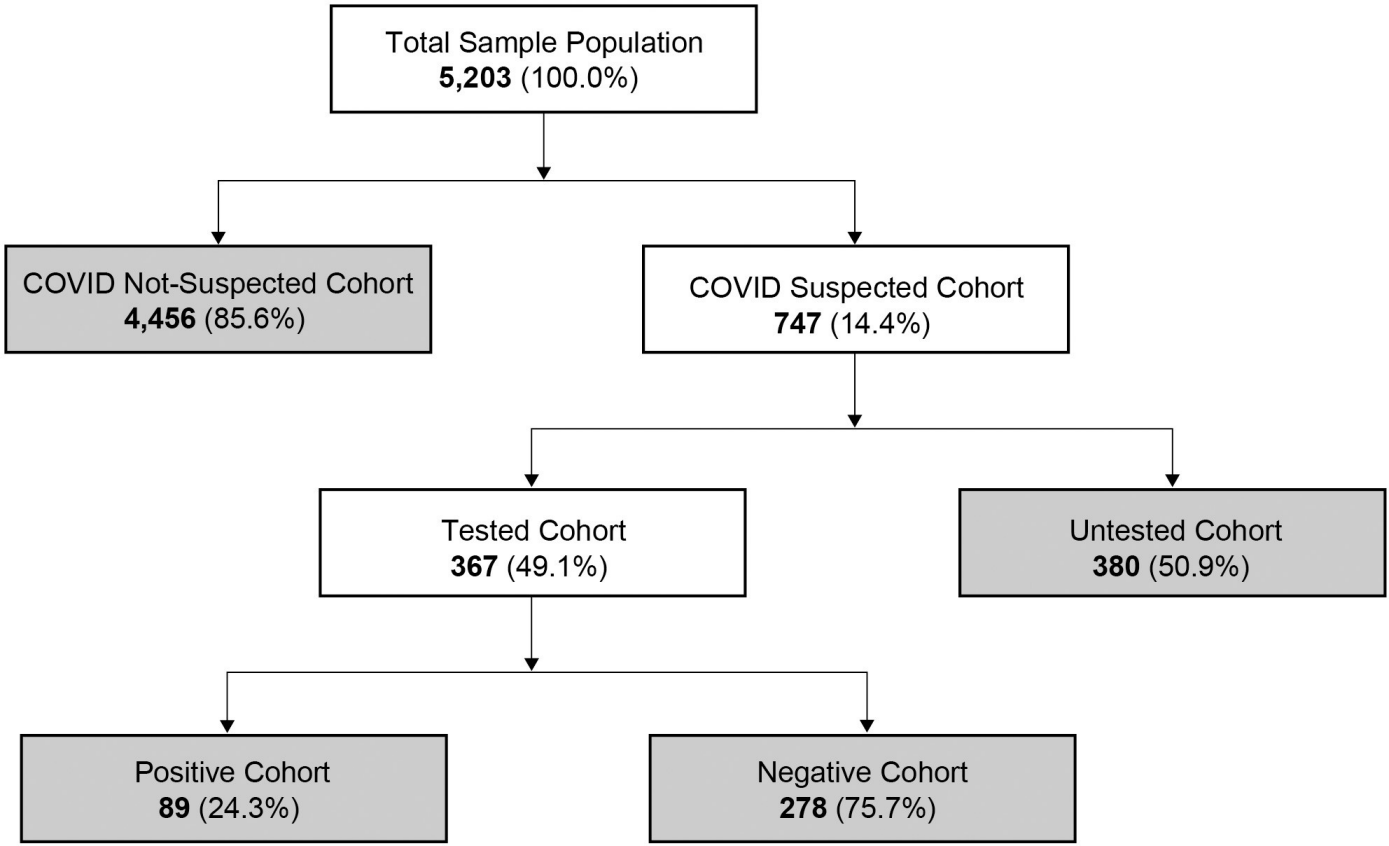
Flow diagram of study participants.

The majority (85.6%) of the 5,203 participants reported no COVID-19 symptoms (COVID Not-suspected Cohort) and 747 (14.5%) reported COVID-19 symptoms (COVID Suspected Cohort) (Fig 1). In the COVID Suspected Cohort, 367 (49.1%) received a diagnostic test (Tested Cohort). In the Tested Cohort, 89 (24.3%) participants reported testing positive for COVID-19 (Positive Cohort) and 278 (75.7%) reported testing negative (Negative Cohort). Approximately 51% of the patients who suspected they had COVID-19 were not tested (Untested Cohort, n = 380). Of the total population, only 1.7% reported testing positive for COVID-19.

Gender, age, race, and comorbidities varied across cohorts (Table 1). Overall, there was a higher proportion of males and younger-aged individuals in both tested cohorts, and higher comorbidities among participants who reported being tested. There was a higher proportion of males in both the tested cohorts (Positive and Negative at 67% and 73%, respectively) compared with the other cohorts. Mean age was among the lowest for both tested cohorts (Positive and Negative Cohorts at 37 and 39 years old, respectively) compared with the other two cohorts (Suspected-not-tested and Untested at 43 and 49 years old, respectively, *p*<0.001).

Significant differences were observed across the cohorts for ethnicity (*p* = 0.001). Although only 10.1% of the sample identified their ethnicity as Black or African American, 20.2% were in the Tested Positive Cohort compared with the other three cohorts (Tested Negative, 8.3%; Untested, 6.1%; COVID Not-suspected Cohort, 10.3%; *p*<0.001). In contrast, 76.9% of the sample who identified their ethnicity as White were more likely to be in the Tested Negative Cohort (84.5%) and less likely to be in the Tested Positive Cohort (68.5%; *p*<0.001).

The CCI score was highest for the Positive Cohort compared with the other cohorts (mean [SD] = 1.02 [1.78] for Positive vs 0.53 [1.18] for Negative Cohort; *p* = 0.003). The prevalence of respiratory related comorbidities (chronic obstructive pulmonary disease [COPD], chronic bronchitis, or emphysema) was significantly higher among the Positive Cohort compared with the Negative Cohort (6.7% vs 1.8%; *p* = 0.017, respectively). Additionally, the Positive Cohort reported more hospitalizations compared with the Negative Cohort (27.0 vs 16.9%; *p* = 0.037).

In this study, 7.8% (n = 407) reported receiving a serological test. In the Positive Cohort, 56 participants received a serological test, of which 64.3% (n = 36) reported testing positive for antibodies. In the Negative Cohort, 141 reported having a serological test, of which 15.6% (n = 22) reported testing positive for antibodies.

### Symptoms and severity of symptoms

**Fig 2:**
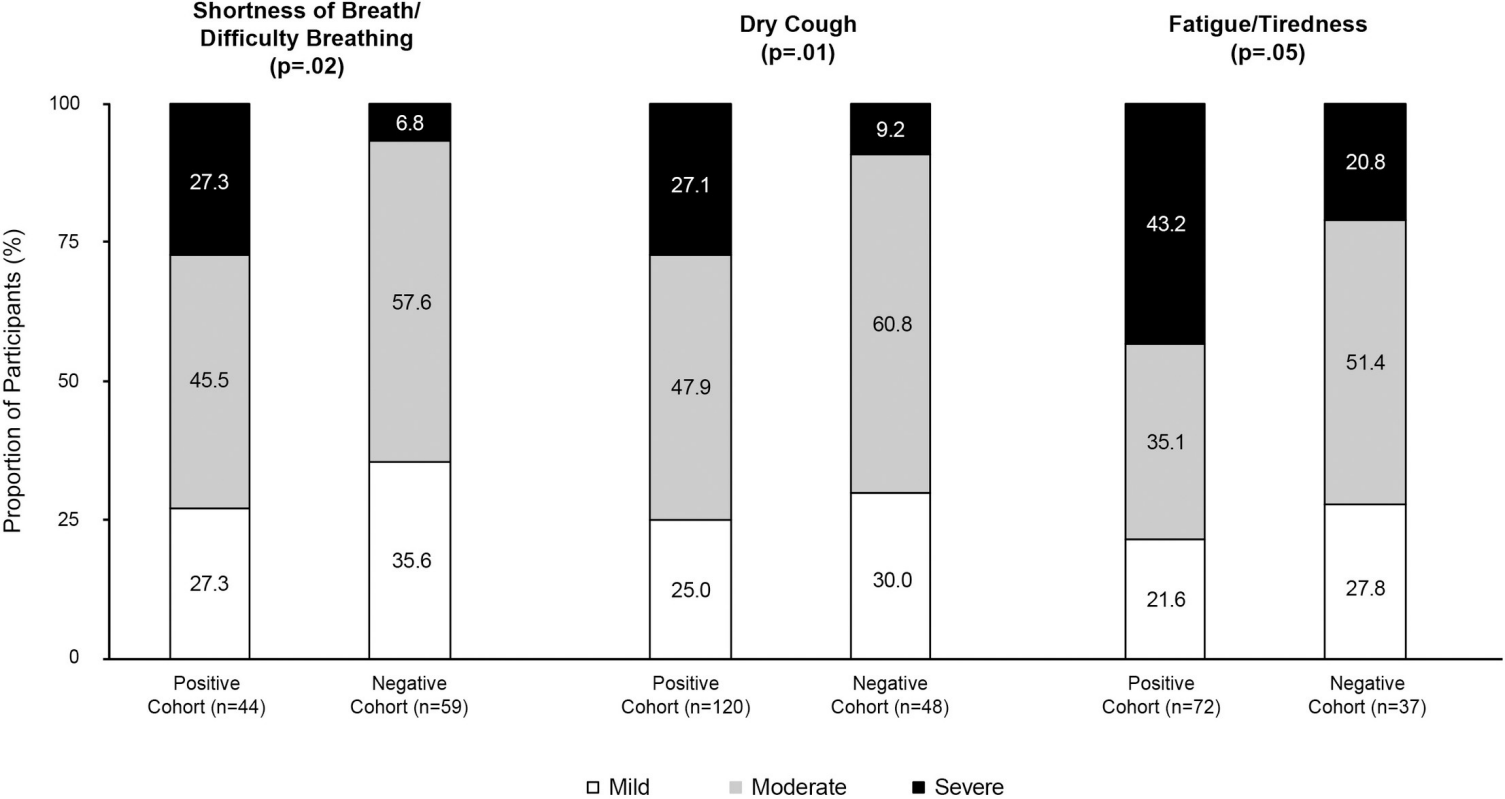
Severity of symptoms commonly associated with COVID-19.

The top three COVID-19 related symptoms in the Positive Cohort were fever (57.3%), dry cough (53.9%), and shortness of breathy/difficulty breathing (49.4%). For the Negative Cohort, the top three symptoms were fever (49.6%), headache (44.2%), and dry cough (42.8%). Shortness of breath/difficulty breathing, respiratory issues, fatigue/tiredness, new loss of smell and taste, diarrhea, persistent pain or pressure in the chest, chills and shaking, and trouble waking up after sleeping were significantly lower for the Negative Cohort compared with the Positive Cohort (Table 2). The severity of the symptoms of shortness of breath/difficulty breathing, dry cough, or fatigue/tiredness was significantly greater in the Positive Cohort compared with the Negative Cohort (*p* = 0.018, *p* = 0.011, and *p*<0.048, respectively) (Fig 2).

**Table 2:** Frequency of COVID-19-related symptoms for positive and negative cohorts.

Symptom	Positive Cohort (n = 89) No. (%)	Negative Cohort (n = 278) No. (%)	P-value
Fever	51 (57.30)	138 (49.64)	0.208
Dry cough	48 (53.93)	119 (42.81)	0.067
Shortness of breath/difficulty breathing	44 (49.44)	59 (21.22)	<0.001
Headache	40 (44.94)	123 (44.24)	0.908
Fatigue/tiredness	37 (41.57)	72 (25.90)	0.005
Respiratory issues	33 (37.08)	37 (13.31)	<0.001
Muscle pain	29 (32.58)	62 (22.30)	0.051
Sore throat	24 (26.97)	83 (29.86)	0.602
New loss of smell and taste	24 (26.97)	23 (8.27)	<0.001
Body aches and pains	22 (24.72)	69 (24.82)	0.985
Chills	22 (24.72)	44 (15.83)	0.057
Nasal congestion	20 (22.47)	57 (20.50)	0.691
Diarrhea	16 (17.98)	27 (9.71)	.035
Persistent pain or pressure in chest	16 (17.98)	20 (7.19)	0.003
Runny nose	15 (16.85)	65 (23.38)	0.194
Chills and shaking	14 (15.73)	22 (7.91)	0.031
Nausea	14 (15.73)	36 (12.95)	0.506
Trouble waking up after sleeping	14 (15.73)	14 (5.04)	0.001
Toe sores or a rash on the toe(s)	5 (5.62)	7 (2.52)	0.152
Conjunctivitis (pink eye)	2 (2.25)	5 (1.80)	0.788
Bluish color to lips or face	2 (2.25)	6 (2.16)	0.960
Other	1 (1.12)	0 (0.00)	0.077

### Treatment management

Ninety-six percent of the participants in the Positive Cohort and 93.9% in the Negative Cohort reported receiving antimalarial drugs, azithromycin, antivirals, or other prescription medications. Compared with those who reported testing negative, the Positive Cohort reported greater use of azithromycin (35.3% vs 18.8%; *p* = 0.002). Rates of antimalarial drugs (41.2% vs 36.4%; *p* = 0.430), antivirals (32.9% vs 23.4%; *p* = 0.080), or other prescription medications (17.6% vs 20.7%; *p* = 0.542) were similar between groups.

### Mental and physical health impact

Measuring mental and physical health with the PROMIS^©^ global health questionnaire allowed a comparison of scores to national norms (ie, 50 for both mental and physical health). Mental health component scores were highest for those who tested positive for COVID-19 and recovered and lowest for those who tested positive and are still recovering (51.20 vs 46.11; *p*<0.001). Physical health component scores were highest for those who tested negative for COVID-19 and lowest for those who tested positive and are still recovering (49.90 vs 43.14; *p* = 0.002).

### Factors predictive of positive test results for COVID-19

**Fig 3:**
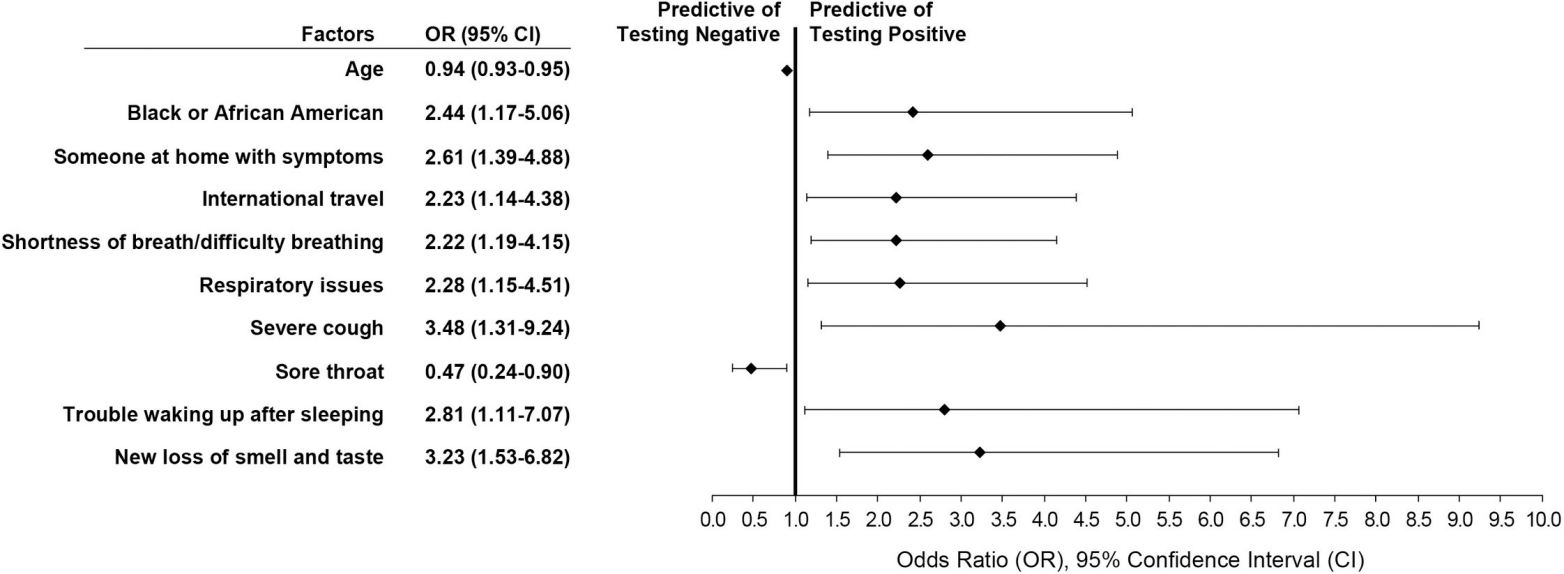
Factors predictive of reporting a positive COVID-19 diagnostic test result. Multivariate regression analysis of factors predictive of a positive test result after controlling for participant reported characteristics (demographics, symptoms, and other risk factors).

The multivariate logistic regression analysis showed that having a severe dry cough (OR 3.482; *p* = 0.012), new loss of taste or smell (OR 3.233; *p* = 0.002), trouble waking up (OR 2.805; *p* = 0.029), someone at home with symptoms (OR 2.607; *p* = 0.003), recent international travel (OR 2.234; *p* = 0.019), the symptoms of respiratory issues (OR 2.279; *p* = 0.018) or shortness of breath (OR 2.222; *p* = 0.012), and ethnicity of Black or African American (OR 2.435; *p* = 0.017) were predictive of testing positive for COVID-19. There was a lower likelihood of testing positive with reported sore throat (OR 0.468; *p* = 0.022) and older age (OR 0.942; *p*<0.001) (Fig 3).

## Discussion

This rapid assessment of symptoms and symptom severity during an early phase of the COVID-19 pandemic provides important insights regarding SARS-CoV-2 infection in a community-based population not widely available in other data sources. In late April 2020 and early May 2020, a large portion of the US was under stay-at-home orders. Access to COVID-19 tests was limited to individuals who met specific criteria, including healthcare professionals, international travel to select high-risk countries, hospitalization with COVID-19-like symptoms, or individuals who were in close proximity to someone diagnosed with COVID-19 [4]. Using data from a large, nationally-representative community sample allowed for real-time reporting of COVID-19 symptoms experienced by adults.

At the time this data was collected only 7.1% of the population surveyed had received a diagnostic test for COVID-19. This study highlights the level of the population with COVID-19 symptoms that were and were not able to be tested. Given the limited access to testing at the time of data collection, the true incidence of COVID-19 was likely much higher than reported. Conversely, the true mortality rate would be lower given the lack of testing. When testing is expanded to a greater number of the US population the incidence and mortality rate will be more precise.

In this study the most common symptoms were dry cough, fever, and shortness of breath/difficulty breathing. Those who tested positive were more likely to report higher symptom severity vs those who tested negative. Respiratory related comorbidities (ie, COPD, chronic bronchitis, or emphysema) were also more common for those that tested positive vs negative. Those in the community with respiratory symptoms or related comorbidities appear to be at greater risk for testing positive.

Factors predictive of testing positive for COVID-19 identified in this study included modifiable and unmodifiable characteristics. Factors most likely to be associated with a COVID-19 positive test result were symptoms (severe cough, shortness of breath/difficulty breathing, respiratory issues, and new loss of taste and smell), Black ethnicity, living in a home with someone experiencing COVID-19 symptoms, or recent international travel. Trouble waking up after sleeping was a predictor of a positive test result and may be a proxy for general fatigue, which has been a commonly reported symptom. Age and sore throat were identified as being predictive of a negative test result. Older individuals who were healthy may have been more likely to participate in this study. At the time of this study, sore throat may have been associated with unrelated conditions (e.g., seasonal allergies). These findings are largely consistent with results recently reported in the literature [9].

In this community-based sample, younger adults were more likely to be tested and report a positive COVID-19 test than other cohorts. These younger adults may have been more likely to congregate in social settings or may have been in regions of the country where stay-at-home orders were less restrictive. Similar to prior studies, individuals with comorbidities, especially respiratory-related comorbidities, seem to be at a higher risk of severe outcomes with SARS-CoV-2 infection [10]. In this study, COVID-19 positivity was disproportionately higher for individuals who identified their ethnicity as Black compared with the total population. Disparities in access to healthcare may be an underlying factor contributing to these findings [11].

In this study, 27% of individuals who reported a positive diagnostic test said they were negative for COVID-19 antibodies, while 16% of the individuals who reported a negative diagnostic test reported a positive COVID-19 antibody test. These discrepancies could be related to various factors including subsequent infection after a diagnostic test, receiving a serological test before sufficient antibody titers are present, or questionable reliability of the serological test. As diagnostic and serological testing for COVID-19 becomes more widely available, our understanding of the true incidence, prevalence, and symptom profile of COVID-19 in the community will become clearer. Given the ongoing threat of COVID-19, it would be interesting to determine if those who report a positive diagnosis for COVID-19 can be re-infected.

Until effective therapies or vaccines become available, there are important strategies we can adopt now to build a stronger immune system and help prevent infection. These include healthy eating, exercise, stress reduction, and ample rest, as well as eating whole foods to ensure micronutrients vitamin C and vitamin D are within the Recommended Dietary Allowance (RDA) levels [12–17]. Studies have shown that raw manuka honey is beneficial for anti-influenza viral activity [18,19]. Adoption of healthy lifestyle measures provides opportunity for building stronger immune systems.

Self-reported survey data is subjective in nature. This survey captured data from participants who are in the community. Patients in the hospital, especially due to severe symptoms, would most likely not participate in a survey of this nature. This survey collected information on live participants only, hence mortality data is not reported. Data has shown increased mortality with older age, therefore the sample could have been skewed with respect to the ability to detect those with higher morbidity or who were hospitalized [20]. This study demonstrated a community-based representative sample can be collected quickly to identify characteristics of individuals most likely to test positive for COVID-19. These factors help identify individuals who should consider taking greater precautions and be prioritized for testing.

## Conclusion

The evolving COVID-19 healthcare crisis has had a significant impact on the public health of the US population. These findings help inform our understanding of the symptoms experienced by individuals in the community who are infected with SARS-CoV-2 and factors associated with increased risk for a positive diagnostic test result. Better epidemiologic data will be accessible as more robust testing and tracing capabilities become available. As we gather more data, more accurate statements can be developed to help guide the management of COVID-19 in the US population. For now, the community should continue to adhere to recommendations from public health authorities, including mask wearing, hand washing, and social distancing.
